# Tetraketide α-pyrone reductases in sporopollenin synthesis pathway in *Gerbera hybrida*: diversification of the minor function

**DOI:** 10.1038/s41438-021-00642-8

**Published:** 2021-10-01

**Authors:** Lingping Zhu, Teng Zhang, Teemu H. Teeri

**Affiliations:** Department of Agricultural Sciences, Viikki Plant Science Centre, University of Helsinki, 00014 UH Helsinki, Finland

**Keywords:** Transgenic organisms, Enzymes

## Abstract

The structurally robust biopolymer sporopollenin is the major constituent of the exine layer of pollen wall and plays a vital role in plant reproductive success. The sporopollenin precursors are synthesized through an ancient polyketide biosynthetic pathway consisting of a series of anther-specific enzymes that are widely present in all land plant lineages. Tetraketide α-pyrone reductase 1 (TKPR1) and TKPR2 are two reductases catalyzing the final reduction of the carbonyl group of the polyketide synthase-synthesized tetraketide intermediates to hydroxylated α-pyrone compounds, important precursors of sporopollenin. In contrast to the functional conservation of many sporopollenin biosynthesis associated genes confirmed in diverse plant species, *TKPR2*’s role has been addressed only in Arabidopsis, where it plays a minor role in sporopollenin biosynthesis. We identified in gerbera two non-anther-specific orthologues of *AtTKPR2*, *Gerbera reductase 1* (*GRED1*) and *GRED2*. Their dramatically expanded expression pattern implies involvement in pathways outside of the sporopollenin pathway. In this study, we show that GRED1 and GRED2 are still involved in sporopollenin biosynthesis with a similar secondary role as AtTKPR2 in Arabidopsis. We further show that this secondary role does not relate to the promoter of the gene, AtTKPR2 cannot rescue pollen development in Arabidopsis even when controlled by the *AtTKPR1* promoter. We also identified the gerbera orthologue of *AtTKPR1*, *GTKPR1*, and characterized its crucial role in gerbera pollen development. GTKPR1 is the predominant TKPR in gerbera pollen wall formation, in contrast to the minor roles GRED1 and GRED2. *GTKPR1* is in fact an excellent target for engineering male-sterile gerbera cultivars in horticultural plant breeding.

## Introduction

Pollen grains of land plants are surrounded by a sculpted pollen wall, which plays critical roles in protecting male gametophytes against various biotic and abiotic stresses and, therefore, in plant sexual reproduction^1^. Pollen wall commonly comprises of a pectocellulosic inner intine layer and a tough outer exine layer mainly composed of sporopollenin^1–3^. Exhibiting remarkable chemical stability and physical strength, sporopollenin has been considered as the most resistant of natural biopolymers, and it is regarded as the major component enabling the resistance of pollen walls to various stresses^3,4^. Sporopollenin has been widely reported in various extant and fossil species across the plant kingdom, including Bryophytes where it is part of the spore wall. Production of sporopollenin is considered to have been a key prerequisite for plants to spread on land^3,4^.

**Fig. 1:**
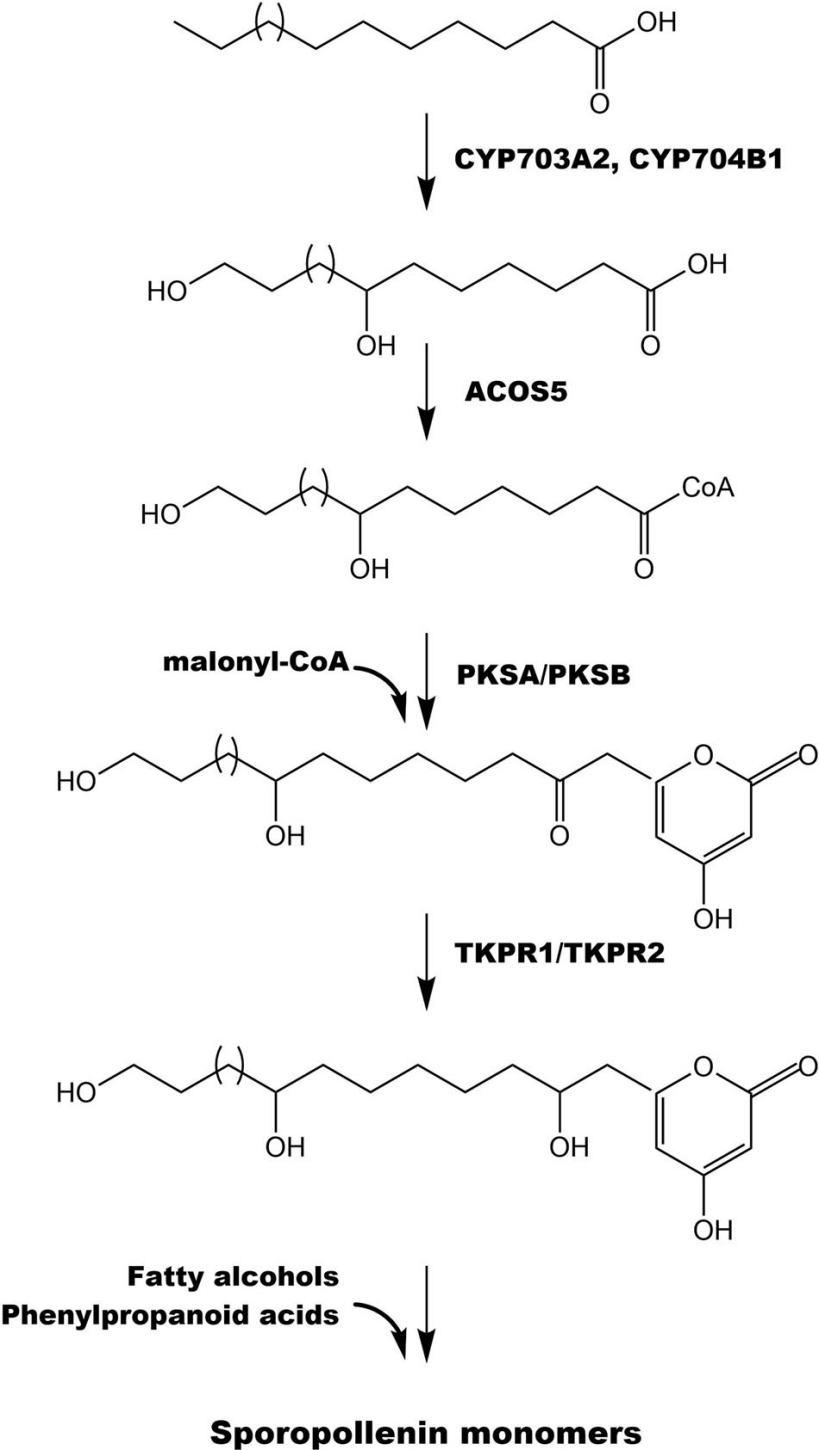
Proposed pathway of sporopollenin precursor biosynthesis in Arabidopsis. Medium- to long-chain fatty acids undergo hydroxylation catalyzed by cytochrome P450-enzymes CYP703A2 and CYP704B1, and are then esterified to CoA thioesters by the fatty acyl-CoA synthetase ACOS5. The generated hydroxyl fatty acyl-CoA esters are subsequently condensed with malonyl-CoA by the chalcone synthase-like polyketide synthases PKSA and PKSB to yield tetraketide α-pyrones, which are further reduced by tetraketide α-pyrone reductases TKPR1 and TKPR2. The resulting polyhydroxylated tetraketide compounds then function as building blocks for sporopollenin

Despite the importance, the chemical composition of sporopollenin has remained obscure due to its biochemically and physically extremely resistant nature. Decades’ attempts by applying multiple degradation studies and nuclear magnetic resonance (NMR) methods have demonstrated that sporopollenin is a complex biopolymer consisting of highly cross-linked hydroxylated aliphatic, aromatic, and phenylpropanoid-derived moieties^2,5,6^. Recent investigations by using thioacidolysis degradation in combination with NMR analysis further revealed aliphatic polyketide-derived polyvinyl alcohol units as the major structural components of sporopollenin and that phenylpropanoid derivatives are essential components that exist as covalently linked structural units^7,8^. Investigations of Arabidopsis mutants with defective pollen exine have facilitated the understanding of sporopollenin composition through genetic and biochemical studies^9–14^. Aliphatic polyhydroxylated tetraketide α-pyrones were revealed to be the important sporopollenin building blocks in these studies^12^. A series of genes encoding key enzymes involved in sporopollenin precursor biosynthesis was identified and characterized in Arabidopsis^9–14^, and a pathway of sporopollenin biosynthesis therefore has been proposed^12,15^ (Fig. 1). In the proposed pathway, medium- to long-chain fatty acids undergo hydroxylation catalyzed by cytochrome P450-enzymes CYP703A2 and CYP704B1, and are then esterified to CoA by a fatty acyl-CoA synthetase (ACOS5), the generated hydroxyl fatty acyl-CoA thioesters being subsequently condensed with malonyl-CoA by the chalcone synthase-like polyketide synthase A (PKSA) and PKSB to yield tetraketide α-pyrones, which are further reduced by tetraketide α-pyrone reductase 1 (TKPR1) and TKPR2 (Fig. 1). The resulting polyhydroxylated tetraketide products function as the building blocks for sporopollenin^8,12^ (Fig. 1). These enzymes have been shown to be specifically expressed in the tapetum of Arabidopsis anthers and tightly co-regulated during pollen development, and their close orthologues have been found widespread in land plants ranging from mosses to angiosperms with conserved male-organ-specific expression patterns^9–14^.

In contrast to Arabidopsis ACOS5, CYP703A2, CYP704B2, PKSA, PKSB, and TKPR1, whose orthologues have been characterized to play conserved functions in sporopollenin biosynthesis in many species including rice^16^, tobacco^16^, and rapeseed^17^, AtTKPR2’s role has been addressed only in Arabidopsis^12^. Both AtTKPR1 and AtTKPR2 were shown to catalyze the same enzymatic reaction in vitro; however, AtTKPR2 seems to play a minor role in sporopollenin biosynthesis^12^. While the *tkpr1* mutant in Arabidopsis makes completely sterile pollen grains with severely damaged exine, the *tkpr2* mutant produces fertile pollen with only mildly impaired exine^12^. Unlike the other sporopollenin biosynthesis associated enzymes that were demonstrated to constitute an endoplasmic reticulum localized metabolon, AtTKPR2 was shown to preferentially localize in the cytoplasm^12,18^.

The ornamental plant *Gerbera hybrida*, from the sunflower family Asteraceae, is one of the economically most important cut-flower crops globally^19^. Gerbera has been worked into a model plant of Asteraceae, and has contributed to flower development and secondary metabolite biosynthesis studies for decades^19^. The gerbera type III polyketide synthases include a small family of the archetypical chalcone synthases^20,21^, but we also identified the first plant pyrone polyketide synthase, gerbera 2-pyrone synthase 1 (G2PS1), that utilizes acetyl-CoA as a starter to initiate the synthesis of two antimicrobial polyketide derivatives, gerberin and parasorboside^22^. Later, we identified two homologues of G2PS1 (G2PS2 and G2PS3) that use the same substrates but catalyze a longer elongation in the biosynthesis of another defense-related polyketide, 4-hydroxy-5-methylcoumarin^23^. Orthologues of Arabidopsis PKSA and PKSB, the gerbera anther-specific chalcone synthase-like 1 (GASCL1) and GASCL2, display the conserved tapetum-specific expression pattern and catalyze the conserved sporopollenin precursor biosynthesis with medium- to long-chain fatty CoA esters as starters^24^. During the exploration of accessory reductases in gerberin and parasorboside biosynthesis, we identified a reductase gene that displays a similar wide expression pattern as G2PS1, *Gerbera reductase 1* (*GRED1*), and its close paralogue *GRED2*. Both *GRED1* and *GRED2* are orthologues of *AtTKPR2*.

The dramatically expanded expression pattern of GRED1 and GRED2 implies involvement in pathways outside the anthers. In this study, we show that GRED1 and GRED2 are nevertheless involved in sporopollenin biosynthesis with a similar secondary role as AtTKPR2 in Arabidopsis. We further show that this secondary role does not relate to the promoter of the gene, AtTKPR2 cannot rescue pollen development in Arabidopsis even when controlled by the *AtTKPR1* promoter. We also identified the gerbera orthologue of AtTKPR1, GTKPR1, and characterized its crucial role in gerbera pollen development. GTKPR1 is the predominant TKPR in gerbera pollen wall formation, in contrast to the minor roles of GRED1 and GRED2. *GTKPR1* is in fact an excellent target for engineering male-sterile gerbera cultivars in horticultural plant breeding.

## Results

### Identification and analysis of orthologous genes for *AtTKPR1* and *AtTKPR2* in gerbera

A single orthologue of *AtTKPR1* was identified in gerbera by a BLAST search^25^ of the gerbera transcriptome using *AtTKPR1* as the query sequence and named *GTKPR1*. *GTKPR1* was recovered in full length from gerbera anther cDNA. The cDNA contains a 990 bp open reading frame (ORF) showing 68% identity with *AtTKPR1* at nucleotide sequence level. *GRED1* and *GRED2* are two orthologues of *AtTKPR2* that were identified and cloned during our exploration of the accessory reductases in the biosynthesis of the defense-related polyketides gerberin and parasorboside. Both *GRED1* and *GRED2* contain a 960 bp ORF and they share 85% nucleotide sequence identity with each other, and 68% and 66% identity with *AtTKPR2*, respectively (Table S2). Further BLAST searches of the gerbera transcriptome did not uncover any additional *AtTKPR2-*like transcripts in gerbera. Pairwise comparisons of the gerbera and Arabidopsis *TKPR1* and *TKPR2* encoded amino acid sequences showed that GTKPR1 and AtTKPR1 showed 73% identity in amino acid sequence, GRED1, GRED2, and AtTKPR2 shared 67% and 65% identity, respectively, while AtTKPR1 and GTKPR1 showed no more than 50% identity with AtTKPR2 or GRED1 or GRED2 (Table S2).

**Fig. 2:**
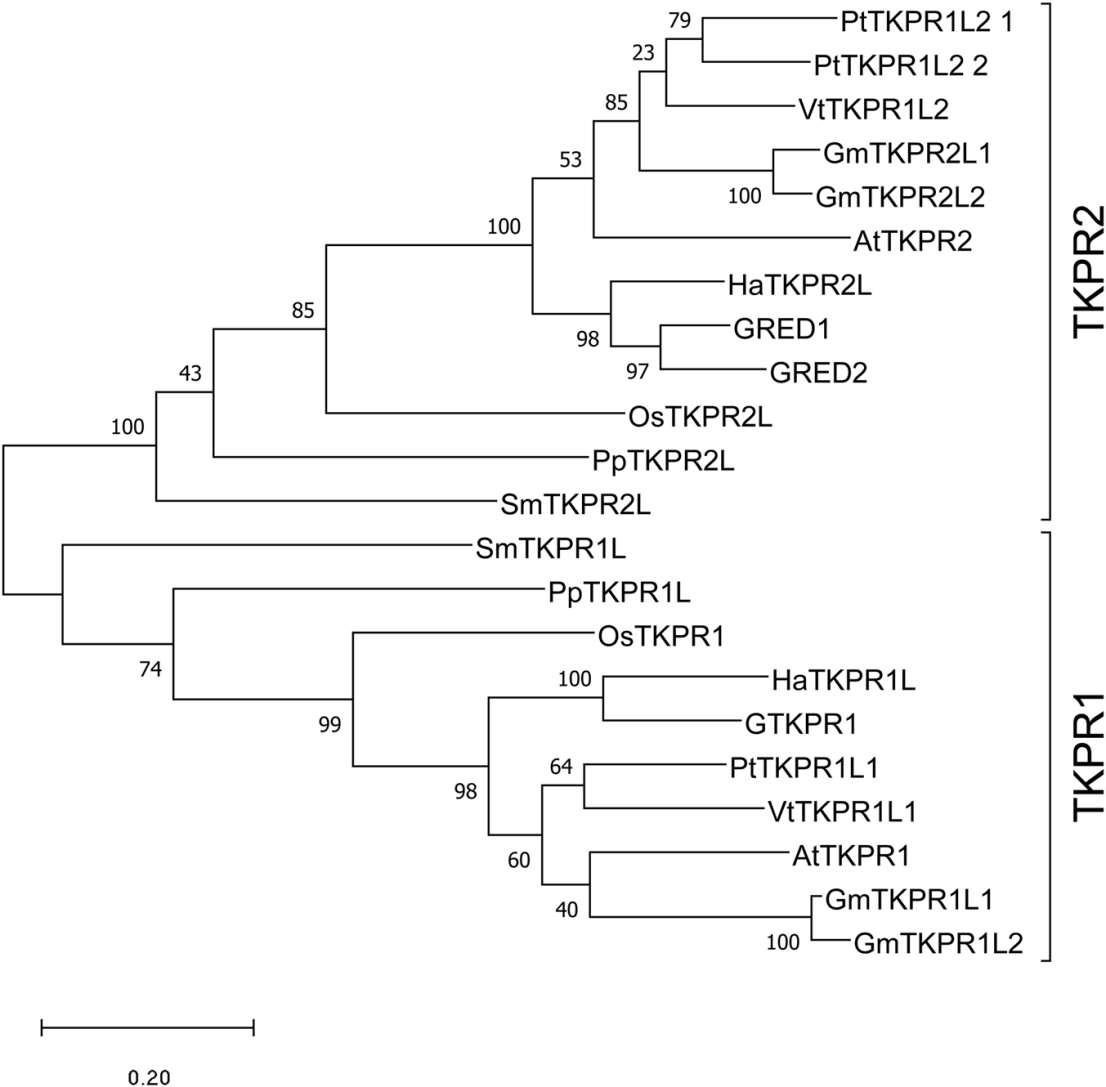
Phylogenetic analysis of *TKPR1* and *TKPR2* sequences from selected species. Full amino acid sequences from *Physcomitrella patens (Pp)*, *Selaginella moellendorffii (Sm)*, *Oryza sativa (Os)*, *Helianthus annuus (Ha)*, *Glycine max (Gm)*, *Populus trichocarpa (Pt)*, *Vitis vinifera (Vv)*, *Arabidopsis thaliana (At)*, *Gerbera hybrida (G)* were aligned and then back-translated to the corresponding original nucleotide sequences. In the unrooted maxiumum-likelihood phylogenic tree *TKPR1*- and *TKPR2*-like genes form separate clades. Bootstrap support values are indicated at the nodes. The scale indicates branch lengths measured in the number of substitutions per site

*AtTKPR1* and *AtTKPR2* orthologues from selected species ranging from mosses to eudicots were extracted from the GenBank database. We found that there was only a single *AtTKPR1* orthologue in all species we analyzed (except in soybean), while some species, like gerbera, had two *AtTKPR2* orthologues. In phylogenetic analysis, the reductases clustered in two clades. *GTKPR1*, *AtTKPR1*, and other *AtTKPR1* orthologues clustered the TKPR1 clade while *AtTKPR2*, *GRED1*, *GRED2*, and the other *AtTKPR2* orthologues formed the TKPR2 clade (Fig. 2).

### *GTKPR1* shows tapetum-specific expression, while *GRED1* and *GRED2* are universally expressed in various gerbera organs but in the anther, they are tapetum-specific

**Fig. 3:**
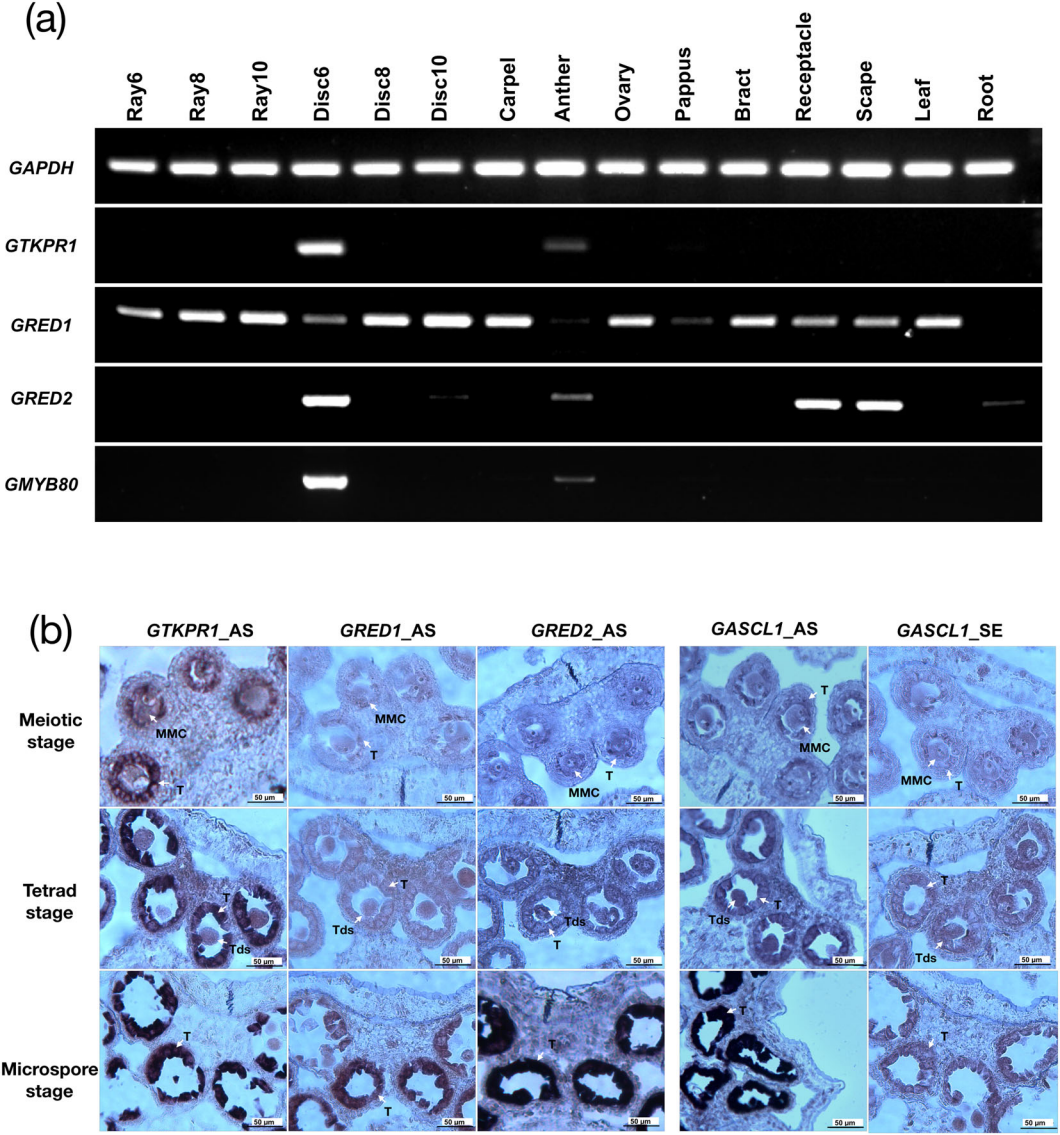
Expression patterns of *GTKPR1*, *GRED1*, *GRED2*, and *GMYB80*. **a** Semi-quantitative RT-PCR analysis of expression levels of *GTKPR1*, *GRED1*, *GRED2*, and *GMYB80* in a series of gerbera tissues. Stage 6 disc flowers have active tapetal cells, the anther sample is a mixture of stages 6, 8, and 10. The constitutively expressed gerbera *GAPDH* gene was used as control. **b** RNA in situ hybridization of *GTKPR1*, *GRED1*, and *GRED2* in developing anthers of stage 6 disc flowers. Sense and anti-sense probes of *GASCL1* were applied as negative and positive controls. MMC microspore mother cells, Tds tetrads, T tapetum

To investigate the expression patterns of *GRED1*, *GRED2*, and *GTKPR1* in gerbera, we analyzed their expression profiles in 15 gerbera tissues by RT-PCR. Our data shows that *GRED1* is highly expressed in various gerbera organs but only moderately expressed in anthers; *GRED2* is preferentially expressed in the disc flowers, anthers, receptacle, scape, and roots (Fig. 3a). Neither of them displayed the anther-specific expression pattern like the Arabidopsis *AtTKPR2*. Unlike the universally expressed *GRED1* and *GRED2*, *GTKPR1* was specifically expressed in disc flowers at inflorescence stage 6 with developing anthers (Fig. 3a), consistent with the reported expression pattern of Arabidopsis *AtTKPR1*, tobacco *NtTKPR1*, and rice *OsTKPR1*^12,16^.

To further investigate the precise locations of *GTKPR1*, *GRED1*, and *GRED2* expression in gerbera anthers, we conducted RNA in situ hybridization experiments with gene-specific antisense probes in sections containing anthers at various developmental stages. An antisense probe for *GASCL1*, the gerbera tapetum-specific orthologue of *AtPKSA* in sporopollenin biosynthesis, was used as a positive control, and its sense probe was used as a negative control. Hybridization signals of the *GTKPR1* probe were exclusively detected in the tapetum cells, starting from the meiosis stage when the tapetum was first visible until the microspore stage when the tapetum degenerated. The strongest signals were observed at the microspore stage, which was consistent with the in situ localization of *AtTKPR1* in Arabidopsis anthers and *NtTKPR1* in tobacco anthers^12,16^ (Fig. 3b). The hybridization signals of *GRED1* and *GRED2* were also exclusively observed in the tapetum cells of the anthers; however, they were only observed at the microspore stage when the microspores were released from the tetrads, and the *GRED1* probe displayed much weaker signals compared with the *GTKPR1* or *GRED2* probes (Fig. 3b). This observation is consistent with the reported *AtTKPR2* expression, which was specifically expressed in tapetum but was more restricted temporally compared with *AtTKPR1*^12^.

Sporopollenin biosynthesis genes are under common transcriptional regulation in Arabidopsis and particularly the transcription factor MYB80 (MS188) is an upstream regulator of all known biosynthetic genes, including *AtTKPR2*^26^. We investigated if the expanded patterns of *GRED1* and *GRED2* expression in gerbera would be the result of expanded expression of the gerbera MYB80 homologue *GMYB80*. Expression of *GMYB80* follows closely the pattern of *GTKPR1*, not *GRED1* or *GRED2* (Fig. 3a) indicating that GMYB80 is not responsible for their expression in vegetative tissues.

### Downregulation of GTKPR1, GRED1, and GRED2 differentially affect gerbera pollen development

**Fig. 4:**
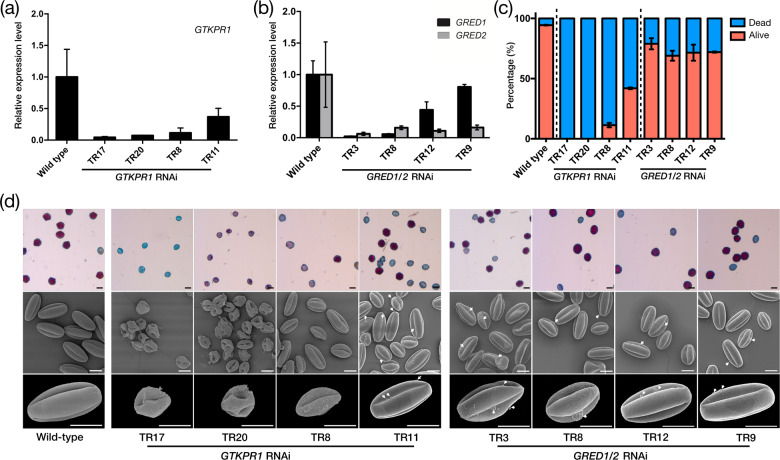
Expression and phenotype analysis of gerbera *GTKPR1*, *GRED1*, and *GRED2* RNAi downregulated transgenic lines. **a**, **b** Expression analysis of severely, moderately, and mildly repressed *GTKPR1* RNAi lines and *GRED1/2* RNAi lines. qRT-PCR shows the expression levels of the corresponding genes in disc flowers of inflorescence development stage 6. **c** Statistics for the normal and dead pollen grains in gerbera wild-type and transgenic lines. **d** Alexander staining and SEM observation of pollen grains made by gerbera wild-type and transgenic lines, arrows refer to damaged exine. Error bars (± SE) are calculated from three biological replicates. Scale bars are 30 μm

To investigate the physiological functions of *GTKPR1* in gerbera pollen grain development, we applied a double-stranded RNA interference (RNAi) vector to produce *GTKPR1* downregulated gerbera transgenic lines. We characterized several lines that showed specific suppression of *GTKPR1* (Fig. 4a) and observed that their inflorescences showed visible phenotypes (Fig. S1). Compared with the wild-type inflorescences displaying healthy anthers and large amounts of released pollen, the severely downregulated lines (*GTKPR1*_RNAi TR17 and TR20) and the moderately downregulated line (*GTKPR1*_RNAi TR8) showed shrunken anthers without any released pollen, and the mildly downregulated line (*GTKPR1*_RNAi TR11) showed normal anthers and a small amount of released pollen grains in the spring (Fig. S1) but not in the winter (not shown in figure). We characterized four lines (*GRED1/2* RNAi TR3, TR8, TR9, and TR12) that showed cross-suppression of both *GRED1* and *GRED2* (Fig. 4b). All four lines grew similar inflorescences as the *GTKPR1* mild suppression line that showed normal anthers and small amounts of release pollen grains in the spring (Fig. S1).

We further applied Alexander staining to examine the viability of pollen grains produced by the transgenic lines and applied SEM to examine the morphology of the pollen grains in detail. The results show that all pollen grains produced by the *GTKPR1* severe suppression lines (*GTKPR1*_RNAi TR17 and TR20) were severely distorted with damaged exine and showed no viability at all (Fig. 4c, d). More than 80% of the pollen grains produced by the *GTKPR1* moderate suppression line (*GTKPR1*_RNAi TR8) were dead and these pollen grains showed a degree of distortion in shape and damage in exine (Fig. 4c, d). The mild suppression line (*GTKPR1*_RNAi TR6) produced around 50% of dead pollen grains based on the staining, and SEM showed that these pollen grains were not distorted but showed a mild exine damage (Fig. 4c, d). The GRED1/2 downregulation lines produced 10% to 30% dead pollen grains that also showed mild exine damage (Fig. 4c, d), a similar phenotype as the pollen grains in the Arabidopsis *tkpr2* mutant^12^. The pollen exine damage was relatively heavier in the severe suppression lines (*GRED1/2* RNAi TR3, TR8), which displayed a mildly distorted shape compared with pollen in the moderated suppression lines (*GRED1/2* RNAi TR9, TR12) that showed a nearly normal shape (Fig. 4c, d). As a control, the wild-type gerbera produces 95% alive pollen grains with a smooth surface and healthy exine (Fig. 4c, d).

### Expression of *GTKPR1* in Arabidopsis *tkpr1* mutant rescues the male-sterile phenotype, while expression of *AtTKPR2, GRED1, and GRED2* do not

**Fig. 5:**
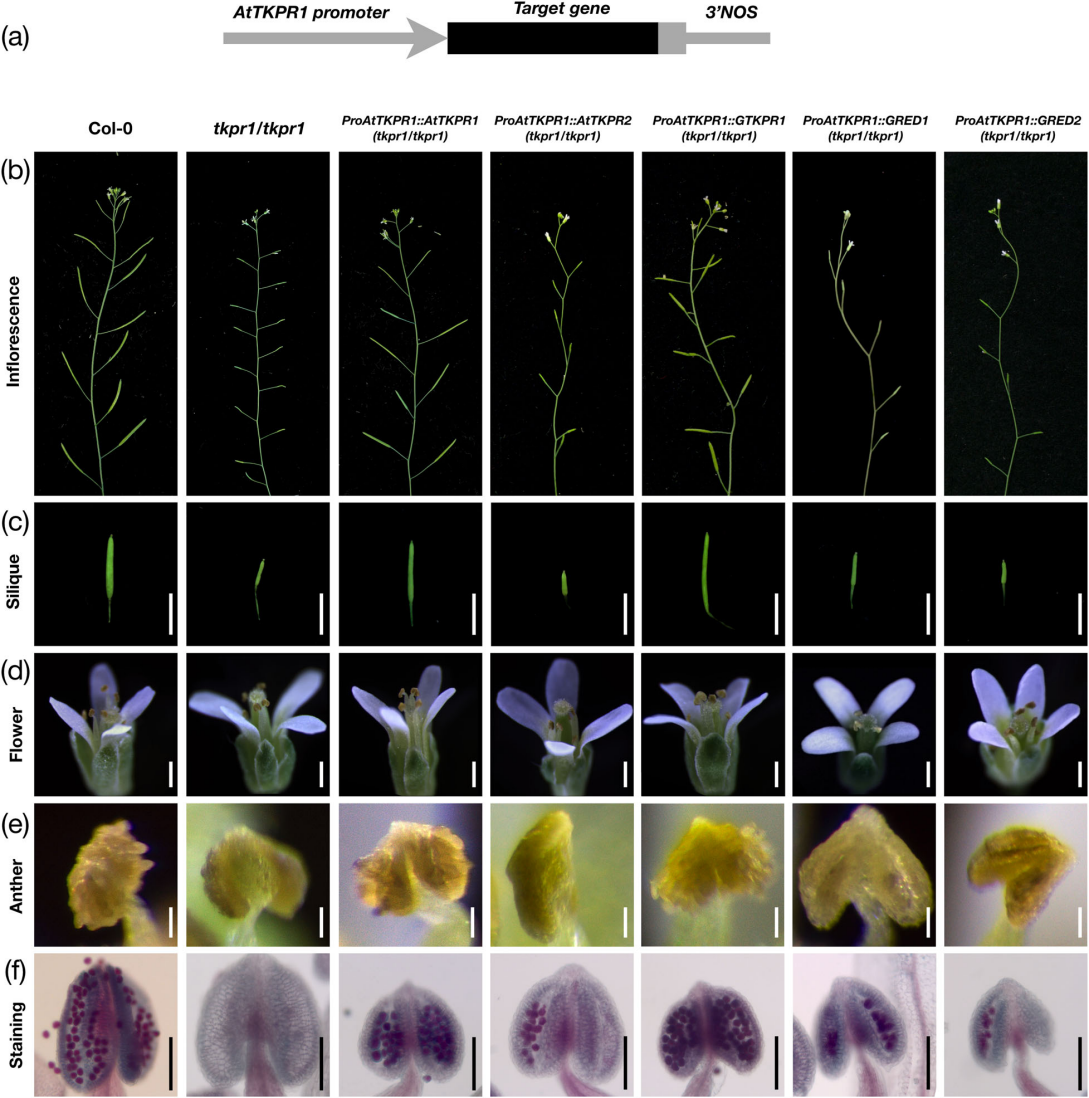
Rescue of pollen development in Arabidopsis. **a** Structural representation of complementation constructs for *AtTKPR1*, *AtTKPR2*, *GTKPR1*, *GRED1*, and *GRED2*, driven by the *AtTKPR1* promoter. **b**–**f** Inflorescences, siliques, flowers, anthers, and viability staining of pollen grains in Arabidopsis nontransgenic plants (Col-0 and *tkpr1* homozygous) and *AtTKPR1*, *AtTKPR2*, *GTKPR1*, *GRED1*, and *GRED2* transgenic plants in *tkpr1* homozygous background. Scale bars are 50 mm in **c**, 500 μm in **d**, and 50 μm in **e** and **f**

AtTKPR1 and AtTKPR2 have been shown to catalyze the same in vitro enzymatic reactions and both were considered to catalyze the reduction of tetraketide α-pyrones in Arabidopsis sporopollenin biosynthesis^12^. To verify that GTKPR1 plays conserved function with AtTKPR1, and to investigate whether AtTKPR2 and its orthologues GRED1 and GRED2 can catalyze the same in vivo functions as AtTKPR1, we performed Arabidopsis mutant complementation experiments. Coding sequences of *GTKPR1*, *AtTKPR2*, *GRED1*, and *GRED2* were independently transformed into the sterile Arabidopsis *tkpr1* homozygous background under the control of the native *AtTKPR1* promoter (Fig. 5a), and the *AtTKPR1* coding sequence (in the same vector) was transformed as a positive control. We conducted the floral dipping transformations on the fertile flower shoots of Arabidopsis *tkpr1/+* heterozygote and selected the transgenic lines by germinating seeds on hygromycin selection medium. The transgenic lines in the *tkpr1* homozygous background were identified through genotyping PCR with specific primers, and RT-PCR with gene-specific primers was applied to verify the expression of the transformed genes (Fig. S2).

Three or more individual transgenic lines in *tkpr1* homozygous background were screened from each construct. By observing the phenotypes of these transgenic lines, we found that the restoration of pollen fertility and seed formation were achieved in *AtTKPR1* and *GTKPR1* transgenic lines, while the *AtTKPR2*, *GRED1*, and *GRED2* transgenic lines displayed a male sterile phenotype, the same as the nontransgenic *tkpr1* homozygous control (Fig. 5b, c). Released pollen grains were found on the mature anther surfaces of *AtTKPR1* and *GTKPR1* transgenic lines in *tkpr1* homozygous background, while the *AtTKPR2*, *GRED1*, and *GRED2* transgenic lines in *tkpr1* homozygous background did not show any released pollen grains on the anther surfaces, a similar phenotype as the *tkpr1* mutant (Fig. 5c, d). However, the pollen viability assay showed tiny amounts of viable pollen grains in few of the mature anthers of *AtTKPR2*, *GRED1*, and *GRED2* transgenic lines in contrast to the *tkpr1* mutant that displayed practically empty anthers (Fig. 5e). This indicates that the viability of pollen grains was partially resorted in these lines.

## Discussion

The structurally robust biopolymer sporopollenin is the major constituent of the exine layer of pollen wall and plays a vital role in plant reproductive success. In Arabidopsis, enzymes responsible for sporopollenin biosynthesis are encoded by a set of anther-specific genes *CYP703A2/CYP704B1, ACOS, PKSA/PKSB*, and *TKPR1/TKPR2*^9–14^. Extensive evidence suggests that sporopollenin biosynthesis is a highly conserved polyketide biosynthetic pathway present in all land plant lineages and was probably associated with the evolution of land plants^10–12^. In contrast to the functional conservation of many sporopollenin biosynthesis associated genes having been confirmed in several plant species like rice, tobacco, and rapeseed, orthologues of *AtTKPR2* have not been studied in other species than Arabidopsis. Gerbera has two orthologues of *AtTKPR2*, *GRED1*, and *GRED2*. They are expressed not only in anthers (like *AtTKPR2*), but also widely in vegetative tissues, possibly indicating recruitment to biochemical pathways outside of the anthers. In this study, we characterized that GRED1 and GRED2 have kept the conserved tapetum-specific expression pattern in anthers and in fact can and do participate in sporopollenin biosynthesis. Concerning the anther domain, GRED1 and GRED2 of gerbera are similar to AtTKPR2 of Arabidopsis. We also characterized the orthologue of *AtTKPR1* in gerbera, *GTKPR1*, and revealed its critical function in sporopollenin biosynthesis. Our results confirm the conservation of the TKPR function in sporopollenin biosynthesis, and that TKPR1s (e.g., AtTKPR1 and GTKPR1) are the predominate enzymes. The roles of TKPR2s (e.g., AtTKPR2, GRED1, and GRED2) are minor, and the strong expression of GRED1 and GRED2 outside anthers in gerbera rises the interesting question of whether the main function of TKPR2 is in fact outside of sporopollenin biosynthesis even in Arabidopsis.

### GRED1, GRED2, and GTKPR1 are involved in gerbera pollen exine formation

Gerbera *GRED1* and *GRED2*, the two orthologues of *AtTKPR2*, encode two reductases showing high sequence similarities with *AtTKPR2*, and *GTKPR1* is the orthologue of *AtTKPR1* (Table S2). TKPRs catalyze the final reduction of the PKSA/PKSB-synthesized tetraketide intermediates to hydroxylated α-pyrone compounds, important precursors of sporopollenin^12^ (Fig. 1). Despite exhibiting the same in vitro enzymatic functions, AtTKPR2 was suggested to play a minor function in sporopollenin biosynthesis compared to AtTKPR1’s predominant role in the pathway. Homozygous *tkpr2* mutant plants produce fertile pollen with mildly impaired exine while the *tkpr1* mutant makes completely sterile pollen grains with a severely damaged exine^12^. In gerbera, although *GRED1* and *GRED2* were expressed in various organs, in anthers they were exclusively expressed in the tapetum at microspore stage, a precisely similar expression pattern as the one reported for *AtTKPR2*^12^ (Fig. 3a, b). This suggests that *GRED1* and *GRED2* may still be involved in sporopollenin biosynthesis in the anthers.

An important question relating to the minor role of AtTKPR2 in sporopollenin biosynthesis is whether this is due to the promoter and expression pattern of the gene. In vitro, AtTKPR2 catalyzes the same reaction as AtTKPR1^12^. In constructs with a 1500 bp promoter fragment of *AtTKPR1* attached to the cDNA of the reductases, we could show that *AtTKPR1* and *GTKPR1* could fully complement the *tkpr1* mutant in Arabidopsis (Fig. 5b, c). However, the *AtTKPR2* construct could not rescue fertility, but still restored viability of the pollen grains to some extent, concluded from the vital staining that marks the pollen grain cytoplasm (Fig. 5b–f). This shows that AtTKPR1 and AtTKPR2 are not functionally equivalent. *GRED1* and *GRED2* (under the *AtTKPR1* promoter) led to a similar partial restoration of pollen viability as *AtTKPR2* (Fig. 5f), indicating a similar function in this context.

The gerbera *GRED1*/*GRED2* cross-downregulated transgenic lines produced 10–30% of dead pollen grains with a mild exine damage (Fig. 4c), a similar phenotype as reported Arabidopsis *tkpr2* mutant^9^. The gerbera *GTKPR1* severely downregulated lines were however completely sterile and produced 100% of distorted pollen grains with severe exine damage, and milder suppressed lines were partially sterile producing around 50% of dead pollen grains with relative milder exine damage (Fig. 4c, d), resembling the Arabidopsis *tkpr1* null mutants and partial mutants^12^. G*TKPR1* exhibited a typical tapetum-specific expression pattern as do various reported sporopollenin biosynthesis associated genes (Fig. 3b), including gerbera *GASCL1* and *GASCL2*^24^. This is highly consistent with *AtTKPR1* expression pattern in Arabidopsis in the tapetum of anthers, ranging from the meiosis stage to microspore stage^12^.

Completely male-sterile and terribly distorted pollen grains were also observed in rice *Ostkpr1* mutant and tobacco *NtTKPR1* downregulation lines, in which the crucial TKPR functions of OsTKPR1 and NtTKPR1 were characterized previously^16^. These observations suggest that all the three reductases are involved in exine formation in gerbera. Similar as AtTKPR1, GTKPR1 is the predominate TKPR in gerbera sporopollenin biosynthesis and its function is irreplaceable in pollen exine formation. Consistent with AtTKPR2, GRED1, and GRED2 are relatively less crucial for this pathway, majority of pollen grains are alive, and the plants are fertile even without their functions.

### Functional conservation and differentiation of tetraketide α-pyrone reductases in land plants

Searching the latest transcriptome data of several model plants, we showed that *TKPR1* genes nearly always appear as single copy with male-organ specific expression pattern while *TKPR2s* are present as two copies with non-anther-specific expression patterns in several species like gerbera, soybean, and poplar (Fig. 2, Table S3). This pattern suggests that while *TKPR1*s are highly functionally conserved in sporopollenin biosynthesis, *TKPR2*s may have differentiated into novel functions in the process of gene duplication during land plant evolution. The functional conservation of TKPR1s has been confirmed by the precisely consistent tapetum-specific expression pattern of AtTKPR1, OsTKPR1, NtTKPR1, and GTKPR1 and consistent severely distorted exine phenotype presented in corresponding mutant plants reported previously and in this work^12,16^. This is also strongly supported by our observation that *GTKPR1* successfully restored the fertility of Arabidopsis *tkpr1* mutant. *TKPR2*s have been rarely studied previously and TKPR2’s minor role in sporopollenin biosynthesis was only characterized in Arabidopsis^12^. Consistent male organ-specific expression pattern in most species suggests that TKPR2s would play conserved functions in sporopollenin biosynthesis. However, the dramatically expanded expression pattern of gerbera *GRED1* and *GRED2*, and of soybean and poplar *TKPR2* genes, implies that they might have acquired novel functions involved in pathways outside the anthers.

### *TKPR1* is a potential molecular target for male sterility in breeding

Male sterility of plants is an important trait with applications in agricultural and horticultural crop breeding, such as effective production of hybrid seeds, guarding of germplasm in commercial ornamental breeding and in preventing of undesired diffusion of seeds or pollen grains produced by genetically modified crops in the open field^27^. In this study, we showed that *TKPR1* is one of the key genes controlling the plant’s male fertility and is highly functionally conserved in land plants. *TKPR1* can be applied as a molecular target for controlling male fertility in crops through modern genetic methods such as RNAi or CRISPR-Cas9^28^. Present as single copy, the gene is a straightforward target with a low off-target risk. In this work, we successfully made male sterile gerbera transgenic lines by suppression of *GTKPR1* expression, demonstrating the application in an important ornamental plant species.

## Materials and methods

### Plant materials

*Gerbera hybrida* cultivar ‘Regina’ was obtained from Terra Nigra B.V, The Netherlands. Wild-type and transgenic gerbera plants were multiplied in vitro and grown as previously described^29^. *Arabidopsis thaliana* (ecotype Col-0) T-DNA insertion line SAIL_837_D01 seeds of a *tkpr1* heterozygous plant were obtained from the Nottingham Arabidopsis Stock Centre (http://arabidopsis.info/). In the progeny, the wild-type, *tkpr1* homozygous, and heterozygous individuals were identified by PCR using gene-specific and T-DNA-specific primers (Table S1). The identified *tkpr1* heterozygous plants were used for floral dipping transformation^30^. For the growth of Arabidopsis, seeds were surface sterilized and stratified for 2 days at 4 °C in the dark, then germinated on MS medium (no antibiotics for control plants and supplemented with 30 mg/L hygromycin B (Sigma) for transgenic lines) at 20 °C under continuous light. Two-week-old seedlings were transplanted to soil and grown at 22 °C under long-day conditions (light/dark cycles of 16 h/8 h). Developmental stages of gerbera inflorescences and Arabidopsis flowers are described by Helariutta et al.^31^ and Smyth et al.^32^, respectively.

### Identification and cloning of *GTKPR1*, *GRED1*, *GRED2*, and *GMYB80*

Using the Arabidopsis *tetraketide α-pyrone reductase 1* (*AtTKPR1*) as the query sequence to blast against the gerbera transcriptome data from 35 gerbera tissues (unpublished data), a *TKPR1*-like reductase encoding transcript was identified and named as *GTKPR1*. The *GTKPR1* sequence was recovered in full length and its full coding sequence was amplified from the gerbera cultivar ‘Regina’ anther cDNA with gene-specific primers (Table S1). *GRED1* and *GRED2* are two *TKPR2-like* genes that were identified and cloned based on *GRED1* having a significantly similar expression pattern as the broadly expressed gerbera 2-pyrone synthase *G2PS1* and *GRED2* being similar in sequence with *GRED1*. *GRED1* and *GRED2* sequences were recovered in full length and their full coding sequences were amplified from the cultivar ‘Regina’ petal cDNA of inflorescence development stage 6 with gene-specific primers (Table. S1). *GMYB80* was identified through significant similarity (84–87% identity, 100% coverage) of the encoded amino acid sequence with MYB80 transcription factors of Asteraceae, e.g., with those of *Lactuca sativa* and *Helianthus annuus* (XP_023740601 and XP_022039565, respectively). GenBank accession numbers for the nucleotide sequences for *GTKPR1*, *GRED1*, *GRED2*, and *GMYB80* are MW842918, MW842919, MW842920, and MZ328313, respectively.

### Phylogenetic analysis

To conduct phylogenetic analysis, putative *TKPR1* and *TKPR2* orthologues from selected plant species were obtained from the GenBank database (https://www.ncbi.nlm.nih.gov/genbank/) by conducting BLASTP searches using AtTKPR1 and AtTKPR2 as queries, and their accession numbers are listed in Table S3. The full-length amino acid sequences were aligned using Clustal Omega software^33^ and then back-translated to the corresponding nucleotide sequences. The generated alignment was used to construct a maximum-likelihood phylogenetic tree in MEGA X^34^ with default parameters, and a total of 1000 bootstrap replicates. Expression patterns of *TKPR1* and *TKPR2* orthologues were searched from published articles, ePlant database or model plant genome databases (Table S3).

### Generation of gerbera RNAi transgenic lines

For the gene silencing construct, the full-length ORFs for *GTKPR1*, *GRED1*, and *GRED2* were cloned into the entry vector pDONR221 through the Gateway BP reaction^35^, and the corresponding RNAi constructs were then generated by LR recombination between the entry clones and the Gateway binary vector pK7GWIWG2D(II)^36^. For transformation, the *GTKPR1*, *GRED1*, and *GRED2* RNAi constructs were electroporated into *Agrobacterium tumefaciens* strain C58C1(pGV2260)^37^, and then used for the agrobacterium-mediated transformation of the cultivar ‘Regina’, performed as described earlier^38^.

### Arabidopsis complementation experiment

A 1.5 kb region upstream of the *AtTKPR1* initiation codon was Gateway cloned from Arabidopsis genomic DNA into the entry vector pDONRP4-P1r (Invitrogen) using the primers listed in Table S1 to generate the promoter entry vector pPro_*TKPR1*_entry. The pEnNosT2-R2R3 vector containing a nopaline synthase 3′-terminator flanked by attR2 and attL3 sites was a gift from Ari Pekka Mähönen (University of Helsinki, Finland). Full-length coding sequences of At*TKPR1* and At*TKPR*2 were PCR amplified from Arabidopsis Col-0 flower bud cDNA using gene-specific primers (Table S1) and further cloned into pDONR221. The complementation constructs for *AtTKPR1*, *AtTKPR2*, *GTKPR1*, *GRED1*, and *GRED2*, driven by the *AtTKPR1* promoter, were constructed through Multisite Gateway LR reactions between the pPro_*TKPR1*_entry, entry clones of the target genes, pEnNosT2-R2R3 vector, and the destination vector pHm43GW, according to the manufacturer’s instructions (Invitrogen). The generated complementation constructs were electroporated into *Agrobacterium tumefaciens* strain GV3101(pMP90)^39^ and further transformed independently into the Arabidopsis heterozygous *tkpr1*/+ mutant line by floral dipping^30^. The harvested seeds were screened on MS medium supplemented with 30 mg/L hygromycin B (Sigma-Aldrich) to select the transgenic plants. The transgenic plants in *tkpr1* homozygous background (*tkpr1/tkpr1)* were identified by PCR as described above.

### Gene expression analysis

Semiquantitative RT-PCR was performed to analyze gene expression in gerbera tissue series and in Arabidopsis nontransgenic and transgenic lines. Quantitative real-time PCR (qRT-PCR) was performed to analyze gene expression analysis in gerbera wild-type and transgenic lines. For determination of the expression patterns of *GTKPR1*, *GRED1*, and *GRED2* in gerbera, a series of 15 vegetative and reproductive tissue series were sampled from gerbera cultivar ‘Regina’ for total RNA isolation, including ray and disc flowers from inflorescence development stage 6, 8, and 10, pooled bract, carpel, anther, ovary, pappus, receptacle, and scape samples from inflorescence development stages 6, 8, and 10, pooled leaf sample from 5–8 cm, 25 cm, and 37–38 cm long leaves, and root. For verification of RNAi-efficiency in *GTKPR1* RNAi and *GRED1/2* RNAi gerbera transgenic lines, disc flowers at inflorescence development stage 6 were collected from wild-type, five independent *GTKPR1* RNAi lines (TR8, TR11, TR17, and TR20) and four independent *GRED1/2* RNAi lines (TR3, TR8, TR9, and TR12). For verification of the expression of transformed genes in Arabidopsis, flower buds at stage 9 from transgenic lines and nontransgenic controls were collected for RNA isolation. The total RNA isolation and cDNA synthesis was performed as described previously^24^. For RT-PCR, gene-specific primers (Table S1) were used to amplify the corresponding cDNA sequences under the following PCR program: 95 °C for 3 min, followed by 28 cycles of 95 °C for 30 s, 58 °C for 30 s, and 72 °C for 1 min, and followed by 72 °C for 8 min. qRT-PCR was performed with gene-specific primers (Table S1) as described previously^24^ and relative expression was calculated by using the ΔΔCt method as described by Pfaffl^40^. The gerbera housekeeping gene *GAPDH* and Arabidopsis *TUBULIN* were used as reference genes for gerbera and Arabidopsis, respectively.

### RNA in situ hybridization

The 1–5 outermost rings of disc flowers of the cultivar ‘Regina’ at inflorescence development stage 6 were collected for RNA in situ hybridization, performed as described by Kontturi et al.^24^. Gene-specific fragments for *GRED1* (199 bp), *GRED2* (240 bp), and GTKPR1 (212 bp) were PCR amplified from the *GRED1, GRED2*, and *GTKPR1* entry clones with primers containing the T7 polymerase promoter sequence (Table S1), and then applied to synthesis of digoxigenin-labeled antisense probes following the manufacturer’s protocols of DIG RNA labeling kit (Roche). Gene-specific sense and antisense probes for *GASCL1* synthesized previously^24^ were used as the positive and negative controls.

### Pollen grain viability assay

Optimized Alexander staining described by Peterson et al.^41^ was applied to analyze the viability of pollen grains in gerbera and Arabidopsis transgenic lines. Anthers of gerbera wild-type, *GTKPR1* RNAi, and *GRED1/2* RNAi transgenic lines at inflorescence development stage 9, and flowers of Arabidopsis wild-type, *tkpr1* homologous mutant, and transgenic lines at floral stage 14–15 were collected and fixed in 60% ethanol, 30% chloroform, and 10% glacial acetic acid (V/V) for 2–3 hours, then stained with 2–4 drops of stain solution (0.01% Malachite green, 0.05% Acid fuchsin, 0.005% Orange G, 10% ethanol, and 4% glacial acetic acid) on a slide with a moderate rate of heating until the stain solution was nearly boiling. Rinsed with water and the stained pollen grains were released by pressing slightly with a cover slip. Slides were examined and photographed using the LeitzLaborlux S Microscope (Wetzlar, Germany) equipped with a Leica DFC420 C Digital Camera. Cytoplasm of viable pollen grains is stained in purple while aborted and empty pollen grains stain blue. The alive and dead pollen grains from each gerbera sample were counted from three independent fields with 20–100 pollen grains each.

### Scanning electron microscopy (SEM)

Surface morphology of gerbera pollen grains was examined by SEM. For sampling, flowers were collected from wild-type and selected transgenic lines, at similar developmental stages as compared to those used for pollen grain staining. Pollen grains were squeezed out from the anthers, and directly mounted to carbon double-sided tape supported by a sample stub. Platinum coating was done to all mounted samples by a Quorum Q150TS high-resolution coater (Quorum Technologies, UK). The samples were then imaged by a Quanta 250 SEM device (FEI, Hillsboro, OR, USA) located at the electron microscopy unit, University of Helsinki, Finland. In close-up images of individual pollen grains, background was removed in Adobe Photoshop CC in order to better illustrate the pollen structures.

## Supplementary information

Supplement

Supplementary information

Data Set 1
