# Supplementary material for: Tetraketide α-pyrone reductases in sporopollenin synthesis pathway in Gerbera hybrida: diversification of the minor function
Source: Hortic Res. 2021 Oct 1;8:207. doi: 10.1038/s41438-021-00642-8 (PMC8484347; doi:10.1038/s41438-021-00642-8)
Supplement: Supplementary file 1 — Supplement [file 41438_2021_642_MOESM1_ESM.pdf]

Tetraketide α-pyrone reductases in sporopollenin synthesis pathway in *Gerbera hybrida*: Diversification of the minor function

Lingping Zhu, Teng Zhang and Teemu H. Teeri

Supplementary information

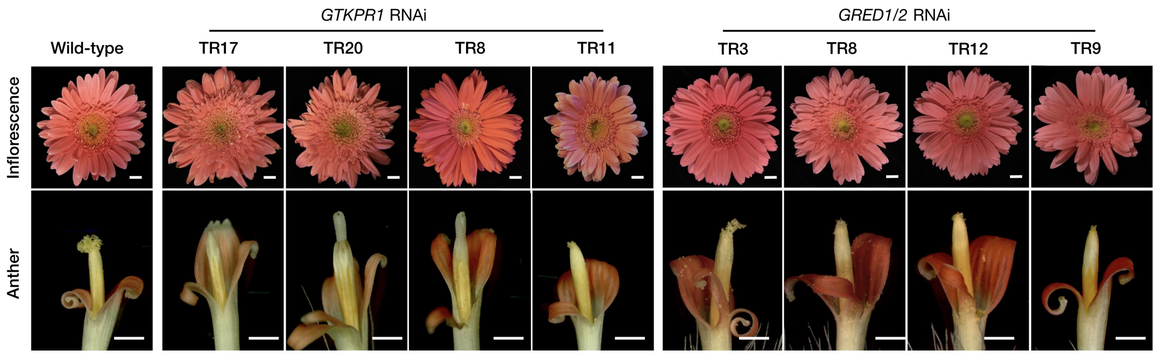

Fig. S1 Inflorescences and anthers of gerbera wild-type, *GTKPR1*, *GRED1* and *GRED2* downregulated gerbera transgenic lines. Inflorescences and anthers are at inflorescence development stage 10. *GTKPR1* downregulated lines do not release pollen while *GRED1/2* downregulated lines occasionally do (in the spring). Scale bars are 10 mm in inflorescences and 1 mm in anthers.

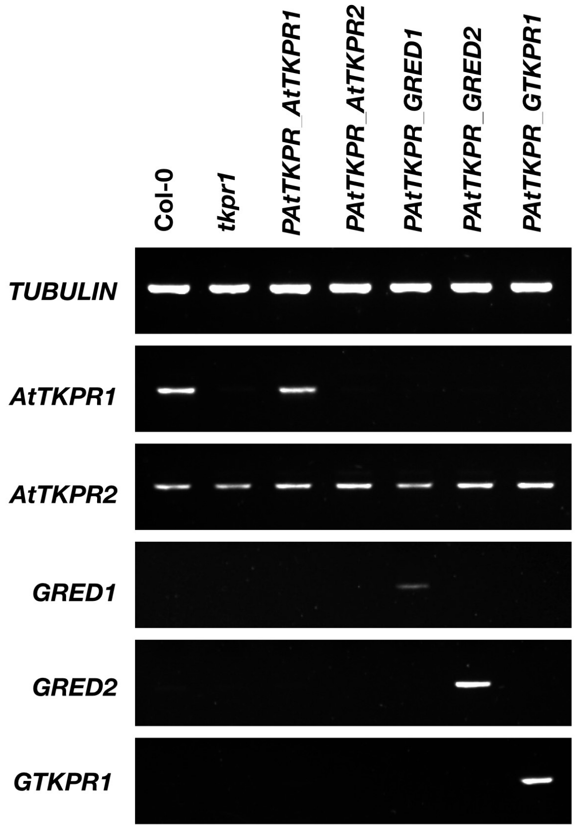

Fig. S2 Expression of transgenes in arabidopsis. Semi-quantitative RT-PCR analysis of expression levels of *AtTKPR1*, *AtTKPR2*, *GTKPR1*, *GRED1* and *GRED2* in arabidopsis nontransgenic plants (Col-0 and *tkpr1* homozygous) and *AtTKPR1*, *AtTKPR2*, *GTKPR1*, *GRED1,* and *GRED2* transgenic plants in *tkpr1* homozygous background. *Arabidopsis TUBULIN* gene was used as the control for constitutive expression.

Table S1 Pairwise comparisons

**Identity through pairwise comparisons of nucleotide sequences**					
	AtTKPR1	*AtTKPR2*	*GTKPR1*	*GRED1*	*GRED2*
AtTKPR1	100%				
AtTKPR2	54.1%	100%			
GTKPR1	68%	52.7%	100%		
GRED1	53.9%	68.5%	52.1%	100%	
GRED2	55%	66.1%	53.4%	85.5%	100%
**Identity through pairwise comparisons of amino acid sequences**					
	AtTKPR1	AtTKPR2	GTKPR1	GRED1	GRED2
AtTKPR1	100%				
AtTKPR2	46.7%	100%			
GTKPR1	73.4%	44.8%	100%		
GRED1	49.5%	67.3%	46.4%	100%	
GRED2	48.9%	65.7%	47.9%	83.8%	100%

Table S2 Genes and expression information for phylogenetic analysis.

Species	Gene name	Gene ID	Expression	Sources
Gerbera hybrida	*GTKPR1*	MW842918	Tapetum	This work
	GRED1	MW842919	Most tissues	This work
	GRED2	MW842920	Receptacle, scape, anther, root	This work
Physcomitrella patens	*PpTKPR1L*	PHYPA_020324	Green sporophyte	PEATmoss[1]
	PpTKPR2L	PHYPA_011652	Green sporophyte	PEATmoss[1]
Selaginella moellendorffii	*SmTKPR1L*	LOC9647944	Strobili	ePlant[2]
	SmTKPR2L	SELMODRAFT_135301	Strobili	ePlant[2]
Oryza sativa	*OsTKPR1*	Os09g0493500	Tapetum	Wang et al.[3]
	OsTKPR2L	Os01g0127500	Inflorescence	ePlant[2]
Helianthus annuus	*HaTKPR1L*	HannXRQ_Chr17g054428	Stamen	ePlant[2]
	HaTKPR2L	HanXRQ_Chr17g0552051	Stamen	ePlant[2]
Glycine max	*GmTKPR1L1*	GLYMA_15G018500	Green pods, root	ePlant[2]
	GmTKPR1L2	GLYMA_13G355600	Flower, young leaf	ePlant[2]
	GmTKPR2L1	GLYMA_07G157200	Root	ePlant[2]
	GmTKPR2L2	GLYMA_01G080700	Root, green pods	ePlant[2]
Populus trichocarpa	*PtTKPR1L*	POPTR_008G138600	Male catkins	ePlant[2]
	PtTKPR2L1	POPTR_010G125400	Xylem	ePlant[2]
	PtTKPR2L2	POTRI_008G120200	Young leaf	ePlant[2]
Vitis vinifera	*VtTKPR1L*	VIT_00023841001	Inflorescence	ePlant[2]
	VtTKPR2L	VIT_01s0011g03480	Inflorescence	ePlant[2]
Arabidopsis thaliana	*AtTKPR1*	AT4G35420	Tapetum	Grienenberger et al.[4]
	AtTKPR2	AT1G68540	Tapetum	Grienenberger et al.[4]

1. https://peatmoss.online.uni-marburg.de/ppatens_db/pp_search_input.php
2. http://bar.utoronto.ca/eplant/
3. Wang Y, Lin Y-C, So J, Du Y, Lo C. Conserved metabolic steps for sporopollenin precursor formation in tobacco and rice. Physiol Plant 2013; 149: 13–24.
4. Grienenberger E, Kim SS, Lallemand B et al. Analysis of TETRAKETIDE α-PYRONE reductase function in arabidopsis thaliana reveals a previously unknown, but conserved, biochemical pathway in sporopollenin monomer biosynthesis. Plant Cell 2010; 22: 4067–4083.

Table S3 Primer sequences used in this study.

Gene	Primer	Sequence
**Forward and reverse primers for Gateway entry clones**		
GTKPR1	*GTKPR1*_attB_F	AAAAAGCAGGCTCG ATGGACCATATTGATGAA
	*GTKPR1*_attB_R	AGAAAGCTGGGTC CTAAGGCATAGAAAGATG
GRED1	*GRED1*_attB_F	AAAAAGCAGGCTTCATGCCGGAATATTGTGTGAC
	*GRED1*_attB_R	AGAAAGCTGGGTCTCACAGAAATCCCTTTTCTT
GRED2	*GRED2*_attB_F	AAAAAGCAGGCTTCATGCCGGAATACTGTGTCAC
	*GRED2*_attB_R	AGAAAGCTGGGTCTCACAGAAATCCCTTTTCTT
AtTKPR1	*AtTKPR1*_attB_F	AAAAAGCAGGCTCG ATGGATCAAGCAAAGGGA
	*AtTKPR1*_attB_R	AGAAAGCTGGGTC TTATGGAAGAACAGTAGATAA
AtTKPR2	*AtTKPR2*_attB_F	AAAAAGCAGGCTCG ATGTCTGAGTATTTGGTA
	*AtTKPR2*_attB_R	AGAAAGCTGGGTC TTAGAGCAGACCCTTCTTC
Promoter of AtTKPR1	*Pro_AtTKPR1*_ attB_F	GGGGACAACTTTGTATAGAAAAGTTGCCGCATTTCTTTGGCTTAGGAT
	*Pro_AtTKPR1*_ attB_R	GGGGACTGCTTTTTTGTACAAACTTG CTTTCCGGTATAAATGGAAT
**Forward and reverse primers for *in situ* hybridization**		
GASCL1	*GASCL1*_SE_F	CATAATACGACTCACTATAGGGGGCAACTCTTCAGCAACTAGG
	*GASCL1*_SE_R	GGATGTGGCCATGAAAGAGA
GASCL1	*GASCL1*_AS_F	GGCAACTCTTCAGCAACTAGG
	*GASCL1*_AS_R	CATAATACGACTCACTATAGGGTCTCTTTCATGGCCACATCC
GRED1	*GRED1*_AS_F	GAACATGGCCATACCGTTCGAG
	*GRED1*_AS_R	CATAATACGACTCACTATAGGGTTGTATGGGACAAGTACCGGAGAC
GRED2	*GRED2*_AS_F	GTCGTTGTGAACCCTGCGTAC
	*GRED2*_AS_R	CATAATACGACTCACTATAGGGCATTTGCAAGACCTCCGACCAA
GTKPR1	*GTKPR1*_AS_F	AGACGGGTGGTTTTCACCTC
	*GTKPR1*_AS_R	CATAATACGACTCACTATAGGGAACGAGGGAAGAACGGTCAC
**Forward and reverse primers for semi-quantitative RT-PCR**		
GAPDH	*GAPDH*_RT_PCR_F	CCAGGAACCCAGAGGAGATACC
	*GAPDH*_RT_PCR_R	GGAGCGGATATGATGACCTTCTTG
GTKPR1	*GTKPR1*_RT_PCR_F	AGACGGGTGGTTTTCACCTC
	*GTKPR1*_RT_PCR_R	AACGAGGGAAGAACGGTCAC
GRED1	*GRED1*_RT_PCR_F	GAACATGGCCATACCGTTCGAG
	*GRED1*_RT_PCR_R	TTGTATGGGACAAGTACCGGAGAC
GRED2	GRED2_RT_PCR_F	GTCGTTGTGAACCCTGCGTAC
	GRED2_RT_PCR_R	CATTTGCAAGACCTCCGACCAA
GMYB80	*GMYB80*_ RT_PCR_F	GATGTGGGAAGAGTTGTAGGTTAAG
	*GMYB80*_ RT_PCR_R	ACTATAGGATTCATAAGATCATTCGAAGG
TUBULIN	*TUBULIN*_RT_PCR_F	GTGGAGCCTTACAACGCTACTT
	*TUBULIN*_RT_PCR_R	GACAGCAAGTCACACCAGACAT
AtTKPR1	*AtTKPR1*_RT_PCR_F	TCCTCGGATTACTGAAAG
	*AtTKPR1*_RT_PCR_R	GGGATAGGGAGTGATGG
AtTKPR2	*AtTKPR2*_RT_PCR_F	AACTGTCCGAAACCCAC
	*AtTKPR2*_RT_PCR_R	TCACATTTGTTGTACCCT

Forward and reverse primers for quatitative RT_PCR		
GTKPR1	*GTKPR1*_qPCR_F	AGACGGGTGGTTTTCACCTC
	*GTKPR1*_qPCR_R	AACGAGGGAAGAACGGTCAC
GRED1	*GRED1*_qPCR_F	GAACATGGCCATACCGTTCGAG
	*GRED1*_qPCR_R	TTGTATGGGACAAGTACCGGAGAC
GRED2	*GRED2*_qPCR_F	CGCACTAGCAACACACAAAC
	*GRED2*_qPCR_R	CTAGCAGCGATTTGATGAGGTA
**Forward and reverse primers for Arabidopsis genotyping**		
AtTKPR1 (SAIL_837_D01)	*LB*	ATTTTGCCGATTTCGGAAC
	tkpr1_LP	GATGCCAAGGAGTGTTCCAT
	tkpr1_RP	TGGACCCAAAAACGAGTCAT
